# Quantifying carbon stocks in shifting cultivation landscapes under divergent management scenarios relevant to REDD+

**DOI:** 10.1002/eap.1764

**Published:** 2018-07-25

**Authors:** Joli R. Borah, Karl L. Evans, David P. Edwards

**Affiliations:** ^1^ Department of Animal and Plant Sciences University of Sheffield Western Bank Sheffield S10 2TN United Kingdom

**Keywords:** carbon sequestration, fallow period, greenhouse gas emission, payments for ecosystem services, secondary forest regeneration, slash and burn

## Abstract

Shifting cultivation dominates many tropical forest regions. It is expanding into old‐growth forests, and fallow period duration is rapidly decreasing, limiting secondary forest recovery. Shifting cultivation is thus a major driver of carbon emissions through deforestation and forest degradation, and of biodiversity loss. The impacts of shifting cultivation on carbon stocks have rarely been quantified, and the potential for carbon‐based payments for ecosystem services (PES), such as REDD+, to protect carbon in shifting cultivation landscapes is unknown. We present empirical data on aboveground carbon stocks in old‐growth forest and shifting cultivation landscapes in northeast India, a hotspot of threatened biodiversity. We then model landscape‐level carbon stocks under business‐as‐usual scenarios, via expansion into the old‐growth forest or decreasing fallow periods, and intervention scenarios in which REDD+ is used to either reduce deforestation of primary or secondary forest or increase fallow period duration. We found substantial recovery of carbon stocks as secondary forest regenerates, with a 30‐yr fallow storing about one‐half the carbon of an old‐growth forest. Business‐as‐usual scenarios led to substantial carbon loss, with an 80% reduction following conversion of old‐growth forest to a 30‐yr shifting cultivation cycle and, relative to a 30‐yr cultivation landscape, a 70% reduction when switching to a 5‐yr cultivation cycle. Sparing old‐growth forests from deforestation using protected areas and intensifying cropping in the remaining area of shifting cultivation is the most optimal strategy for carbon storage. In areas lacking old‐growth forest, substantial carbon stocks accumulate over time by sparing fallows for permanent forest regeneration. Successful implementation of REDD+ in shifting cultivation landscapes can help avert global climate change by protecting forest carbon, with likely co‐benefits for biodiversity.

## Introduction

Deforestation and forest degradation in the tropics contribute significantly to biodiversity loss and generate 12% of global annual anthropogenic carbon emissions (van der Werf et al. 2009, Barlow 2016). Shifting cultivation is the dominant land use across 2.6 million km^2^ in the tropics, of which only 6–19% is cleared annually for crop production (Silva et al. 2011). While this provides subsistence for 200–300 million people across 64 developing countries (Mertz et al. 2009, Li et al. 2014), it is also a major driver of carbon emissions (Fearnside 2000) and biodiversity loss (Ogedegbe and Omoigberale 2011, Ding et al. 2012). Reducing deforestation and forest degradation from shifting cultivation can thus play a key role in averting climate change and the global extinction crisis (Lawrence et al. 1998, Houghton 2012).

Shifting cultivation involves clearing a forest patch using slash‐and‐burn methods. Crops are grown on the cleared land for a few seasons (normally one or two), after which the farmland is left fallow for vegetation regeneration (Mertz 2009). During this fallow period, farmers cultivate other plots and return to clear the regenerated secondary forest in the original plot at the end of the rotation period (Mishra and Ramakrishnan 1983). Historically, the fallow period lasted for 20–30 yr allowing complete regeneration of secondary forest in tropical regions (Rerkasem et al. 2009, Poorter 2016). However, due to increasing human population and more demand for farmland, fallow periods have reduced to just 2–3 yr in many regions, which is insufficient for forest regeneration (Grogan et al. 2012). This causes more frequent rotation in existing shifting cultivation and further clearing of old‐growth forest to compensate for decreasing yield, which leads to carbon emission and biodiversity loss in shifting cultivation landscapes (Raman 2001, Williams et al. 2008, Rossi et al. 2010, Jakovac et al. 2015).

Due to various socioeconomic factors, including human population growth, market development, and government policies, there is an increasing trend of transforming shifting cultivation landscapes to more profitable and intensive land uses, such as cash crop plantations (e.g., rubber; Brookfield et al. 1995) and permanent agriculture (Rerkasem and Rerkasem 1994, De Jong et al. 2001). This trend is particularly evident in tropical Asia (van Vliet 2012), although shifting cultivation is still widely practiced in remote mountains of Bangladesh, Laos, and northeast India (Rasul and Thapa 2003). This transition from shifting cultivation to more intensive land uses can have drastic negative impacts on the environment leading to permanent deforestation and biodiversity loss (van Vliet 2012). Therefore, finding alternative and more sustainable approaches to managing shifting cultivation landscapes is of utmost importance.

Few previous studies have assessed how changes in fallow period or the conversion of primary forest to shifting cultivation affect landscape‐level carbon stocks (Mukul et al. 2016a b). There is an urgent need to do so given the widespread trend for reduced fallow periods (Metzger 2002) and marked expansion of shifting cultivation in recent decades (Castella et al. 2005, Hansen and Mertz 2006, Bogaert et al. 2008, Robichaud et al. 2009). Such assessments are critical to the development of carbon‐based payments for ecosystem services (PES) schemes, such as the “Reducing Emissions from Deforestation and forest Degradation (REDD+)” framework (Mertz 2009). REDD+ provides financial incentives to forest‐rich developing countries for reducing carbon emissions by avoiding deforestation and forest degradation, enhancing forest carbon stocks, and managing forests sustainably (UNFCCC 2010). REDD+ has the potential to avoid deforestation by protecting old‐growth forests from shifting cultivation expansion, avoid forest degradation by maintaining a longer fallow cycle, and to enhance carbon stocks by permanent abandonment of older fallow sites or by moving back from short to long fallow cycles. These approaches might also provide co‐benefits for biodiversity conservation, other ecosystem services and sustainable rural development (Gibbs et al. 2007, Phelps et al. 2012). However, it is not clear which of these REDD+ pathways will maximize carbon storage in a shifting cultivation landscape.

Here, we examine how fallow period affects carbon stocks across regenerating secondary forests following shifting cultivation in Nagaland, northeast India, which is of critical importance for global biodiversity conservation (Myers et al. 2000) and where shifting cultivation occupies nearly three quarters of agricultural area (Pareta 2013). We then use these data to model and compare landscape‐level carbon stocks under two alternative management scenarios of shifting cultivation: (1) “business‐as‐usual” scenarios with reduced fallow periods or expansion into old‐growth forest; and (2) intervention scenarios with efforts to protect forest carbon through mechanisms compatible with REDD+. We assess the relative effectiveness of these scenarios in retaining maximum levels of landscape carbon to identify the optimal allocation of efforts and resources under REDD+ in shifting cultivation landscapes.

## Materials and Methods

### Study area

Our study region comprised three districts (Kiphire, Phek, and Kohima) in Nagaland, northeast India (Appendix S1: Fig. S1) across an altitudinal range of 1,487–2,652 m above sea level (asl; Appendix S1: Table S1). These landscapes are within the Indo‐Burma global biodiversity hotspot and specifically are part of the Fakim Wildlife Sanctuary and Saramati area Important Bird Area (#IN421; BirdLife International 2017). The major forest types of the sampling sites were subtropical broad‐leaved wet hill forests (500–1,800 m asl), subtropical pine forests (1,000–1,500 m asl; to 1,645 m asl in our study area) and montane wet temperate forests (>2,000 m asl; Champion and Seth 1968). Annual rainfall varies from 1,800 to 2,500 mm (Statistical Handbook of Nagaland 2015). Shifting cultivation occupies 71.2% of the total agricultural area in Nagaland (Pareta 2013). Fallow period in this region varies from 6 to 27 yr (J.R. Borah, *personal observation*). Common crops grown in shifting cultivation sites are upland rice (*Oryza sativa*), pearl millet (*Pennisetum glaucum*), maize (*Zea mays*), cassava (*Manihot esculenta*), ginger (*Zingiber officinale*), chili pepper (*Capsicum annuum*), sweet potato (*Ipomoea batatas*), and various pulses (Krug 2009).

### Sampling framework

We sampled in three shifting cultivation landscapes (Kiphire in 2015; Phek and Kohima in 2016), each separated by at least 25 km of mountainous terrain (Appendix S1: Fig. S1). Each landscape comprised shifting cultivation farmland, fallows with regenerating secondary forests (abandoned farmland), and old‐growth forests. Old‐growth forests were sampled as control sites. They had no history of shifting cultivation but had low to moderate levels of disturbance from grazing and selective logging. Under the realistic assumption that adverse anthropogenic activities will not be entirely prevented under REDD+ management scenarios, these old‐growth forests provide a robust estimate of how much carbon could be stored if land currently under shifting cultivation were allowed to regenerate fully and, conversely, the carbon stock that would be lost if shifting agriculture expands into previously unfarmed areas.

We defined the fallow period as the unfarmed interval between cropping periods, during which natural vegetation regenerates. Cropping period (one or two years in our study system) is the duration of cropping at a site following clearing. The entire duration of cultivation, that is, from cropping to the start of the next phase of clearing is termed as a cultivation cycle (cropping period + fallow period). The age of the fallow sites was determined via interviewing the farmers and verified with remote‐sensing data (Appendix S1: Determining the age of secondary forest).

### Carbon sampling

We measured nonsoil carbon stocks across three main habitat types: farmland, secondary forest (accounting for variation in age), and old‐growth forest. We randomly selected 36 400 × 400 m sampling squares across the three habitats in each of the three landscapes (15, 12, and 9 squares in Kiphire, Phek, and Kohima, respectively). The number of squares in each district varied depending on the availability of fallow sites and adjacent old‐growth forest sites (distance between fallow sites to the nearest primary forest across the three landscapes = 2,410.5 ± 1,748 m). Sampling squares were placed at least 300 m apart between different habitats and 400 m apart within the same habitat. Within each sampling square, we located three 10 × 30 m sampling plots (*n* = 108; 3.24 ha sampled in total) that were at least 200 m apart (Appendix S1: Fig. S1B, C, D). We used a large number of relatively small plots across farmland, secondary, and old‐growth forest rather than fewer bigger plots to better capture the small‐scale heterogeneity in land‐use history and topography (altitude and ruggedness; 1,487–2,652 m asl) typical of a shifting cultivation mosaic landscape in the study region (Yadav et al. 2012). Previous studies from such mountainous regions have derived reliable carbon estimates from plots of similar or smaller size: McEwan (2011), 0.04 ha; Zeng et al. (2013), 0.04 ha; Hu et al. (2015), 0.04 ha; Ali et al. (2014), 0.01 ha; Mukul et al. (2016a), 0.025 ha; and Gilroy et al. (2014a), 0.0075 ha. To ensure unbiased selection of plots, we walked 100 m perpendicular from the boundary into the focal habitat type. The resultant end point was used as the first corner of the 10 × 30 m carbon‐sampling plot and the second point was located 30 m to the left (i.e., roughly 30 m parallel to the habitat edge). The other two axes of the rectangular plot were parallel to these two randomly selected points. We followed this methodology consistently for all plots. Within each sampling plot, we first measured aboveground living biomass (trees and lianas) and dead biomass (deadwood and leaf litter) using a composite plot design (Appendix S1: Fig. S1E) and converted these biomass estimates to carbon stocks (see section “Estimating total carbon”).

We did not quantify soil organic carbon as studies from northeast India indicate that soil carbon is resilient to land‐use changes from shifting cultivation and recovers rapidly within the first two years of the fallow period (Lungmuana et al. 2017). In addition, studies from elsewhere in the tropics also suggest that forest age has negligible influence on soil carbon, which accumulates rapidly and then stabilizes following abandonment (Martin et al. 2013, Kotto‐Same et al. 1997).

We took a space‐for‐time substitution approach to assess variation in carbon stock across fallow ages. This approach assumes that the observed spatial sequence truly represents a temporal sequence, such that sites in the sequence differ in age, but are similar in abiotic and biotic components and thus share a similar predictable history of regeneration (Johnson and Miyanishi 2008). To minimize any difference in successional history and thus trajectories of carbon accumulation, we sampled landscapes across similar topography, soil type, and land‐use histories (derived from Landsat images and farmer interviews) as recommended by Walker et al. (2010). We also sampled multiple replicates for younger age classes where variability in vegetation structure is high (Swamy and Ramakrishnan 1987).

#### Estimating live biomass

We determined live biomass by measuring the diameter at breast height (DBH) and wood specific gravity of trees. We measured DBH at 1.3 m from ground level in each 10 × 30 m plot for all trees larger than 5 cm DBH. We measured trees with 1–5 cm DBH in three subplots each of 2 × 2 m in size (T1–T3, Fig. S1E) at 5‐, 15‐, and 25‐m distance from the start of the plot, along the plot midline. To calculate wood specific gravity, we extracted tree cores from all trees larger than 5 cm DBH at 1.3 m with an increment borer (two threads, 5.15 mm diameter, 400 mm bit length; Haglöf, Långsele, Sweden). The full core was placed in water for 30 min to fully hydrate it and the fresh volume (i.e., green volume) was then measured using the water‐displacement method (Chave 2005). Cores were then oven dried at 101°–105°C (Williamson and Wiemann 2010) for 24 h and weighed. Finally, we calculated wood specific gravity (g/cm^3^) from the dry mass (g) to green volume (cm^3^) ratio (Chave 2005):

$$\text{Wood specific gravity}=\frac{\text{wood oven dried mass}}{\text{green volume}}.$$

The extraction of cores was not possible for small trees (1–5 cm DBH), so for these individuals, we used the mean wood specific gravity calculated from large trees within the focal 10 × 30 m plot.

We calculated tree biomass as the mean estimate from suitable allometric equations generated from studies of harvested trees. We used five allometric equations generated for similar forest types to those in our study that incorporated information on DBH and wood specific gravity: two equations for trees in old‐growth forest (Dung et al. 2012, Chave 2014), and three equations for trees in secondary forest (Ketterings et al. 2001, van Breugel et al. 2011, Chave 2014; Appendix S1: Table S2). We did not use equations that included height as a predictor as this is extremely difficult to measure accurately in closed canopy forests and on steep terrain. We did, however, calculate the biomass by measuring heights and DBH of 39 randomly selected trees (DBH range = 75.7–206.9 cm) for which we were able to accurately measure height using a clinometer. For these trees, we compared biomass from the equation that incorporated height with biomass from the one that did not (both equations from Chave 2014). We found that allometric equations with height generated slightly higher biomass estimates than equations without height (matched paired *t* test, *t* = 2.25, *P* = 0.03, RMSE = 6.07 Mg), suggesting that our estimates of biomass are conservative (lower carbon) across our plots. For trees with a DBH of 1–5 cm, we calculated tree biomass using the same allometric equations as those used for larger trees, because the few equations developed specifically for younger trees did not incorporate wood specific gravity as a predictor variable (Nascimento and Laurance 2002).

We measured the DBH at 1.3 m height of all lianas larger than 2 cm DBH in two 1 × 30 m sampling subplots located on the plot sides (V1‐2, Fig. S1E). We converted the liana DBH into biomass using five allometric equations for lianas that have been developed for tropical forests (Putz 1983, Gehring et al. 2005, Schnitzer et al. 2006, Sierra 2007, Addo‐Fordjour and Rahmad 2013, Appendix S1: Table S2). We used the mean of these five estimates as a measure of the biomass of each liana. We calculated subplot liana biomass by summing the biomass estimates of all lianas for each subplot. Finally, liana biomass for each plot was calculated as the average of the two subplot biomass estimates.

#### Estimating dead biomass

We measured deadwood and leaf litter to estimate the carbon stock in dead vegetation in each plot. To estimate deadwood biomass, we recorded all standing and fallen deadwood larger than 5 cm DBH within each 10 × 30 m sampling plot. We measured the diameter at both ends of the fallen dead wood and its total length (in all cases, these measurements were only taken for the section of deadwood inside each plot). For standing deadwood, we measured the diameter at the bottom of the deadwood and its height using either a measuring tape (when the top was accessible) or a clinometer (when the top was not accessible). When possible, we also measured the diameter at the top of the deadwood. We measured deadwood volume using the “frustum of a cone” formula when diameter at the top and bottom could be measured

$$V=\frac{\pi h}{3}\times \left(R^{2}+r^{2}+Rr\right)$$

where *V* is volume (cm^3^), *h* is height/length (cm), *R* is diameter of the base (cm), and *r* is diameter of the top (cm; Pfeifer et al. 2015).

When the top diameter could not be measured, we assessed volume using the formula for the volume of a cone (symbols denote the same parameters as the frustum equation)

$$V=\frac{\pi R^{2}h}{3}.$$

We assigned each standing and fallen deadwood into one of five decomposition classes ranging from class 1 (recently dead intact wood) to class 5 (almost decomposed) following Pfeifer et al. (2015). When deadwood was class 1, we extracted a wood core to calculate deadwood density. For the rest of the decay classes, we extracted wood density estimates for each class from the literature (Pfeifer et al. 2015) to estimate deadwood biomass.

We collected all leaf litter (fallen leaves, twigs, and grasses) from three 1 × 1 m subplots (L1–L3, Fig. S1E) centered within each 2‐m^2^ subplot (T1–T3, Fig. S1E) for each 10 × 30 m plot. We measured total leaf litter volume in situ using a “compression” cylinder (Parsons et al. 2009) and calculated the dry mass (oven dried to constant mass) of a 1 L subsample to estimate total dry biomass of leaf litter.

#### Estimating total carbon

We used our four biomass estimates (living tree, lianas, deadwood, and leaf litter) to calculate biomass within each plot (Mg/ha). To derive an estimate of total carbon stock in each plot, we multiplied the plot‐level biomass estimate by 0.474, which is the wood carbon to biomass ratio for both living and dead carbon estimated by Martin and Thomas (2011).

### Statistical analyses

All analyses were conducted using R 3.3.1 software (R Development Core Team 2017). Prior to analysis, we confirmed that all data used in statistical tests did not violate the assumptions of normality and heteroscedasticity using Shapiro‐Wilk and Levene's tests, respectively. We log_10_‐transformed the carbon estimates prior to analysis to meet the normality assumption of regression analyses. A Moran's *I* test, implemented in the ape package (Paradis et al. 2004) in R software, confirmed that there was limited spatial autocorrelation in total carbon stock and this was not statistically significant (Moran's *I* = 0.082, *P *= 0.08).

#### Variation in carbon stock across habitats and fallow period

We constructed a linear mixed‐effect regression (Lmer) model using the lme4 package (Bates et al. 2015) to examine differences in carbon stocks across the three habitats, that is farmland (*n* = 17 plots), secondary forest (*n* = 55), and old‐growth forest (*n* = 36). We included habitat type and elevation as fixed effects. Similarly, to assess differences in carbon stock across fallow ages of secondary forest, we fitted Lmer models including fallow age and elevation as fixed effects. We included squares nested within landscapes as random intercepts in the model to control for multiple sites within each square. We fitted separate Lmer models for total, living, and dead carbon with the same fixed and random effects. “Elevation” in both model sets was scaled by subtracting the mean and dividing by the standard deviation to facilitate model interpretation (Gelman 2007).

We conducted AIC_c_‐based multimodel inference using the function “dredge” in the MuMIn package in R to run a complete set of models with all possible combinations of the fixed effects including their interaction terms. The function “r.squared” in the same package was used to calculate marginal and conditional *r* ^2^ values for each model, which showed the percentage of variation explained by the fixed and random effects, respectively (Barton 2014). We used an information theoretical approach based on Akaike Information Criterion corrected for small sample sizes (AIC_c_) for model selection. The model with the lowest AIC_c_ value was chosen as the best‐fit model (Burnham and Anderson 2002).

#### Predicting change in landscape‐level carbon under hypothetical scenarios

**Figure 1:**
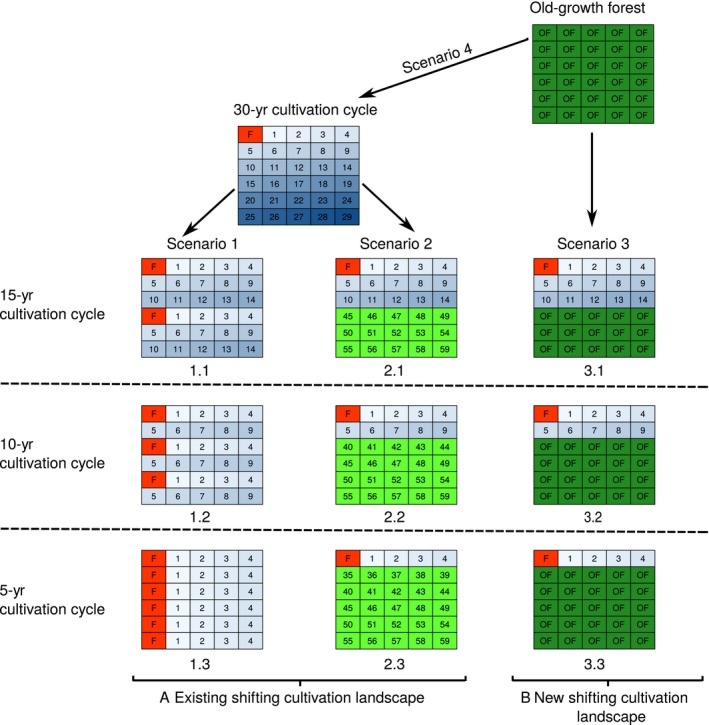
The four sets of management scenarios used to predict changes in landscape carbon in (A) no forest sparing (Scenario 1) and secondary forest creation and sparing (Scenario 2) and (B) new shifting cultivation landscape with old‐growth forest sparing (Scenario 3) and shifting cultivation expansion (Scenario 4). Colors indicate habitat types: farmland (F, red), active fallows (1–29 yr, different shades of blue), abandoned old fallows (>30 yr, light green), and old‐growth forests (OF, dark green). Numbers within cells denote the age of the secondary forests; numbers under cell arrays indicate unique id for each landscape in a scenario.

Our objective was to assess how carbon stocks change under alternative management systems that alter the fallow period in two different types of landscapes: (1) existing shifting cultivation that, at the start, contains farmland and various ages of regenerating secondary forest, but no old‐growth forest (Scenarios 1 and 2, Fig. 1); and (2) pioneer shifting cultivation that, at the start, only contain old‐growth forest (Scenario 3 and 4; Fig. 1). We considered a 5‐yr cultivation cycle as the shortest cycle, because studies show that, with fertilizer inputs, soil fertility restores within the first two years of fallow ensuring a 5‐yr cycle as a viable option for crop cultivation (Thomaz 2013, Lungmuana et al. 2017). We did not include conversion to permanent agriculture in scenario predictions as studies suggest that this is not sustainable in this region, in part due to severe soil erosion and nutrient depletion (Grogan et al. 2012), and there will often also be cultural impediments. We used empirical data from our models of carbon stocks in farmland, secondary, and old‐growth forests to predict landscape‐level carbon stocks under different management scenarios.

Scenario 1 applies to landscapes currently used for shifting cultivation and represents the current trend of decreasing fallow periods to meet growing food demands (no forest sparing, Fig. 1). Thus, it provides a business‐as‐usual scenario without any interventions to reduce carbon emissions. We assume that the initial cultivation cycle is 30 yr (one year of cropping followed by a 29‐yr fallow period), with an equal area of land in each of the 30 possible states, that is farmland and secondary forest of each age class (1–29 yr postfarming). We predicted the change in landscape‐level carbon when increasing demand for food is met by reducing the fallow period but without expanding cultivation to additional old‐growth forests. We estimated carbon stocks when the original 30‐yr cycle is reduced to 15 yr (Scenario 1.1), 10 yr (Scenario 1.2), and 5 yr (Scenario 1.3).

Scenario 2 also applies to a landscape currently used for shifting cultivation with a 30‐yr cultivation cycle. However, in this scenario, financial incentives are available to reduce the amount of land used for shifting cultivation, enabling remaining older fallows to regenerate (secondary forest creation and sparing, Fig. 1). Thus, this scenario reduces carbon emissions by avoiding forest degradation and enhancing forest carbon stocks making it relevant to conservation interventions through REDD+. Under this scenario, as fallow period declines, the older fallows are spared from cultivation by increasing agricultural intensity of a part of the landscape. Increased intensification (such as the use of chemical fertilizers) would enable food production to be maintained despite shorter fallow period (Lungmuana et al. 2017). We estimated carbon stocks when 50%, 67%, and 83% of the landscape were removed from shifting cultivation in 15‐yr (Scenario 2.1), 10‐yr (Scenario 2.2), and 5‐yr cultivation cycles (Scenario 2.3), respectively.

Scenarios 3 and 4 apply to landscapes originally covered by old‐growth forest, but converted to a shifting cultivation landscape (i.e., pioneer shifting cultivation; Mertz 2009). Scenario 3 describes the application of conservation interventions, such as protected areas, that limit further clearing of old‐growth forest and associated carbon emissions for expanding shifting cultivation (old‐growth forest sparing, Fig. 1). This scenario is thus relevant to REDD+ interventions to reduce emission from deforestation. This scenario also requires intensification as increasing land areas are spared from shifting cultivation with declining fallow period. We assessed three alternatives for this scenario: conservation of 50%, 67%, and 83% of the old‐growth forest in 15‐yr (Scenario 3.1), 10‐yr (Scenario 3.2), and 5‐yr cultivation cycles (Scenario 3.3), respectively. Our final scenario (Scenario 4) occurs when old‐growth forest is entirely cleared to create a shifting cultivation landscape, thus providing an additional business‐as‐usual scenario with no REDD+ intervention (shifting cultivation expansion, Fig. 1). The shifting cultivation landscape in Scenario 4 has a 30‐yr cultivation cycle, that is the same cycle as that is used for the baseline situation in Scenarios 1 and 2.

Across all scenarios, landscapes consist of 30 individual and uniform‐sized parcels of land. Each parcel is either under shifting cultivation (farmland or fallow site), permanently abandoned regenerating secondary forest (Scenario 2 only), or old‐growth forest (Scenario 3 and 4). To assess temporal variation in carbon accumulation across scenarios, we estimated landscape‐level carbon after 30 yr (i.e., the maximum fallow period across our scenarios) and after a shorter time frame of 5 yr (Fig. S3), giving a snapshot of changes in carbon stocks following interventions. We calculated landscape‐scale carbon using 1,000 simulations for each scenario. This was achieved by randomly allocating, with replacement, each land parcel an estimated amount of carbon from observed values for farmland and old‐growth forest. For secondary forest, we cannot sample with replacement from observed carbon values for each fallow age as there is insufficient observation for each fallow age. We thus fitted a linear mixed‐effect model of carbon as a function of fallow age (with landscape as a random effect) and sampled with replacement from the range of carbon values generated by the model (i.e., taking 95% confidence intervals of parameter estimates into account) for each age. We then summed the predicted carbon estimates together across the 30 sites to derive the predicted landscape‐level carbon stock for each hypothetical scenario at the end of 5 and 30 yr.

## Results

We measured a total of 3,160 stems (range 1.27–280.36 cm DBH), of which 1,976 (62.5%) were from secondary forest and 1,184 (37.5%) were from old‐growth forest. Stems were absent in our farmland plots. We also measured 128 lianas (75.7%, 24.3%, and 0% in old‐growth forest, secondary forest, and farmland, respectively), 226 standing deadwood stems (32.3%, 44.7%, and 23% in old‐growth forest, secondary forest, and farmland, respectively), and 1491 pieces of fallen deadwood (54.4%, 22.4%, and 23.2% in old‐growth forest, secondary forest, and farmland, respectively).

### Variation in carbon stocks across habitats

**Figure 2:**
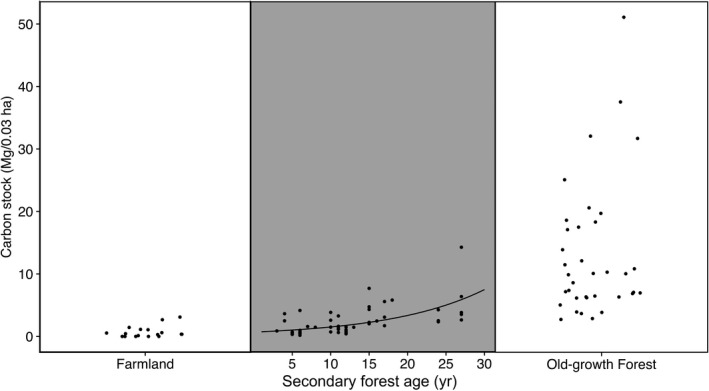
Plots showing total carbon accumulation across the three habitats, farmland, secondary forest with age, and old‐growth forest plots in Nagaland, northeast India. Black line in secondary forest (age in years) shows fitted linear mixed‐effect model.

The best‐fit model for total carbon stock included habitat type as a fixed effect, with higher total carbon in old‐growth forests than secondary forests and farmland (coefficient estimates ± SD, farmland = 0.99 ± 0.13, secondary forest = 1.74 ± 0.08, old‐growth forest = 2.48 ± 0.09; marginal *R* ^2^ = 0.57, conditional *R* ^2^ = 0.76; Fig. 2). For live carbon, the best model included both habitat type and elevation along with an interaction term between habitat type and elevation. This suggests that differences in live carbon stock across habitat types increased with elevation (coefficient estimates ± SD, farmland = 0.01 ± 0.11, secondary forest = 1.39 ± 0.08, old‐growth forest = 2.34 ± 0.09, elevation = 0.06 ± 0.14; marginal *R* ^2^ = 0.81, conditional *R* ^2^ = 0.85; Appendix S1: Fig. S2a). Dead carbon stock showed no significant difference across habitat types (coefficient estimates ± SD, farmland = 1.10 ± 0.13, secondary forest = 1.14 ± 0.08, old‐growth forest =1.58 ± 0.09; marginal *R* ^2^ = 0.19, conditional *R* ^2^ = 0.68; Appendix S1: Fig. S2b).

### Variation in carbon stocks with fallow period

Total carbon stock increased exponentially with fallow age (coefficient estimate ± SD = 0.04 ± 0.01, marginal *R* ^2^ = 0.37, conditional *R* ^2^ = 0.64; Fig. 2), with 30‐yr old fallow sites retaining 56.1% of the carbon stock (7.44 ± 0.32 Mg/0.03 ha) recorded in old‐growth forest (13.24 ± 1.80 Mg/0.03 ha). Live carbon stock showed a similar trend (coefficient estimate ± SD = 0.05 ± 0.01, marginal *R* ^2^ = 0.51, conditional *R* ^2^ = 0.65; Appendix S1: Fig. S2a), but fallow age was not significantly associated with the amount of dead carbon (Appendix S1: Fig. S2b)

### Landscape‐level carbon under alternative management scenarios

**Figure 3:**
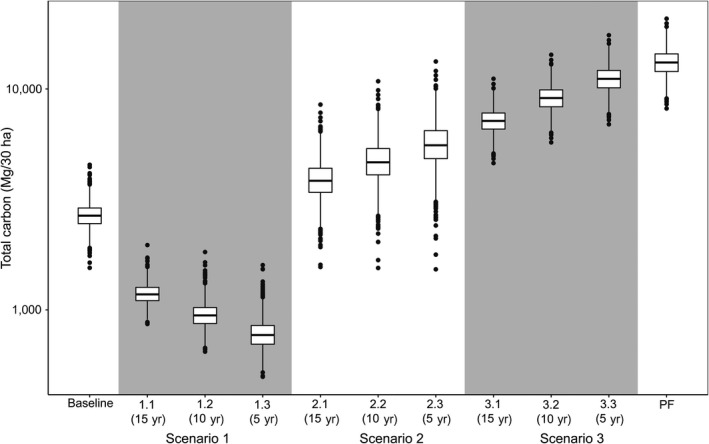
Box plots showing the difference in landscape‐level carbon stock under three alternative management regimes of shifting cultivation at the end of 30 yr relative to a baseline of 30‐yr cultivation cycle (Baseline) and old‐growth forest landscape (OF): (1)“business‐as‐usual” with no forest sparing in Scenario 1 (Scenario 1.1, 15‐yr cycle; Scenario 1.2, 10‐yr cycle; Scenario 1.3, 5‐yr cycle); (2) intervention scenarios by secondary forest creation and sparing in Scenario 2 (Scenario 2.1, 15‐yr cycle; Scenario 2.2,10‐yr cycle; Scenario 2.3, 5‐yr cycle), and old‐growth forests sparing in Scenario 3 (Scenario 3.1, 15‐yr cycle; Scenario 3.2, 10‐yr cycle; Scenario 3.3, 5‐yr cycle). Box plot components are mid line, median; box edges, upper and lower quartile; whiskers, interquartile range; and points, outliers.

Under the business‐as‐usual scenario of no forest sparing (Scenario 1), carbon stocks reduced by 56.3%, 64.8%, and 71% from the 30‐yr baseline of 2,699.7 ± 378.6 Mg/30 ha (mean ± SD) in a 15‐, 10‐, and 5‐yr cycle, respectively (Scenario 1.1, 1.2, and 1.3; Fig. 3). Under the second business‐as‐usual scenario of shifting cultivation expansion (Scenario 4), 79.6% of the carbon stocks in the original old‐growth forest landscape (13,261.7 ± 1,799.7 Mg/30 ha [mean ± SD]) is lost.

In landscapes with already shortened fallow cycles (Scenario 1.3), REDD+ interventions can be applied for enhancement of carbon stocks by converting it from short to long fallow system. For instance, converting the 5‐yr fallow cycle to 10‐, 15‐, and 30‐yr cycles (from Scenario 1.3 to baseline scenario, Fig. 1) enhanced landscape carbon by 21.8%, 51.3%, and 246.4%, respectively. Applying REDD+ style interventions by secondary forest creation and sparing (Scenario 2) also increased carbon stocks substantially. Relative to a 30‐yr baseline landscape, these interventions increased carbon stocks by 46.4%, 77.8%, and 112.4% in 15‐, 10‐, and 5‐yr cycles, respectively (Scenario 2.1, 2.2, and 2.3, respectively; Fig. 3).

In pioneer shifting cultivation landscapes, intervention by old‐growth forest sparing (Scenario 3; Fig. 3) reduced substantial carbon loss compared to the complete conversion of old‐growth forest to a shifting cultivation landscape (shifting cultivation expansion, Scenario 4). Sparing 50% of old‐growth forest (Scenario 3.1) reduced carbon loss by 83.6% relative to a landscape managed entirely as shifting cultivation with a 15‐yr cycle (Scenario 1.1). Similarly, protecting 83% of old‐growth forest (Scenario 3.3) reduced carbon loss by 93% relative to a landscape managed entirely as shifting cultivation with a 5‐yr cycle (Scenario 1.3).

Overall, intervention by old‐growth forest sparing (Scenario 3) held the maximum amount of landscape carbon (54.5%, 69.1%, and 84.3% carbon of an old‐growth forest landscape in Scenario 3.1, 3.2, and 3.3, respectively), followed by secondary forest creation and sparing (29.7%, 36.1%, and 43.1% carbon in Scenario 2.1, 2.2, and 2.3, respectively). Maintaining a longer fallow cycle at 30 yr also retained considerable amount of landscape carbon (20.3%). REDD+ intervention to convert from a short to long cultivation cycle sequestered the least amount of carbon (Scenario 1; 8.9% and 7.2% in 15‐ and 10‐yr cultivation cycle, respectively) when compared to an old‐growth forest landscape.

The above estimates of changes in landscape‐level carbon stocks are calculated at 30 yr following the intervention. Carbon stocks showed similar patterns but less clear differences across scenarios after 5 yr of management changes (Appendix S1: Fig. S3). Intervention scenarios of old‐growth forest sparing retained the highest amount of landscape carbon followed by secondary forest creation and sparing after 5 yr (See Appendix S1: Changes in carbon stocks five years after management changes for more details).

To test if high carbon estimates for old‐growth forests have resulted in an overestimation of the benefits of sparing old‐growth forest relative to those of secondary forest creation and sparing, we reran the simulations replacing our randomly selected primary forest carbon estimates with the median carbon estimates (which is a more conservative estimate being lower than the mean value and thus the value typically used in the random selection process) and with estimates from three comparable published studies that report lower values, that is Mukul et al. 2016a (321.29 Mg/ha), Joshi et al. 2013 (355.09 Mg/ha), Zhang et al. 2013 (376.6 Mg/ha). These studies were selected for comparison as the carbon stocks were estimated from old‐growth forests in (sub‐) tropical mountainous regions in Asia with minimal anthropogenic disturbances, which is similar to our study system. Simulation results (Appendix S1: Fig. S5) show that even with the more conservative estimate (median instead of mean) of primary forest carbon from our study and estimates from other comparable studies, our conclusions on the most optimal scenarios under REDD+ do not change.

## Discussion

Finding an effective way to manage shifting cultivation without adversely affecting crop production is essential for climate change mitigation and biodiversity protection in forest‐rich developing countries. Our study suggests that sparing old‐growth forests by intensifying cultivation in a smaller area (Scenario 3) is the most optimal strategy under REDD+ in (sub‐) tropical forests in mountainous areas. This scenario retained the maximum level of landscape carbon across all business‐as‐usual and intervention scenarios. In existing shifting cultivation, REDD+ can enhance forest carbon by secondary forest creation and sparing (Scenario 2), which stored almost one‐half of the landscape carbon compared to an old‐growth forest. Maintaining a longer fallow cycle and moving from a short to long cultivation cycle also retained a considerable amount of landscape carbon (Scenario 1). Each of these scenarios is particularly relevant under the REDD+ mechanism for reducing carbon emission through avoided deforestation (Scenario 3), avoided forest degradation (Scenario 1, from 5‐yr to 10‐, 15‐, 30‐yr cycles), and conservation and enhancement of forest carbon stock (Scenario 2). Thus, these scenarios illustrate the strong potential of REDD+ for protecting and enhancing forest carbon in shifting cultivation landscapes.

### Carbon stock across habitat types

Although subtropical forests with diverse vegetation contribute considerably to the world's forest carbon stores (Lin et al. 2012), few studies have quantified carbon stocks in old‐growth forests of the subtropics (Ngugi et al. 2014). We show that old‐growth forests in our study area held the highest amount of aboveground carbon (441.4 ± 60 Mg/ha) compared to other habitat types (i.e., farmland and secondary forest). This estimate of old‐growth forest carbon is comparable to the carbon estimates reported from old‐growth forests of Garhwal Himalayas in India (Joshi et al. 2013). However, old‐growth forest carbon estimates from our study area are relatively higher than those reported by other studies from subtropical forests in India (Baishya et al. 2009) and elsewhere (Zhang et al. 2013, Mukul et al. 2016a). The relatively higher carbon estimates in our study can likely be attributed to the low levels of anthropogenic disturbance in the old‐growth forests due to the remoteness and inaccessibility of the region, thus avoiding market‐driven large‐scale forest exploitation. Previous studies from similar sites in India that report lower carbon estimates also reported high levels of anthropogenic disturbances in their old‐growth forest sites, including selective logging/timber extraction (Shaheen et al. 2008, Baishya et al. 2009).

Old‐growth subtropical hardwood forests with minimal anthropogenic and environmental disturbances can accumulate very high levels of biomass as shown in tropical sites from South‐East Asia (McEwan 2011). The relatively undisturbed forests in our study site contained extremely large trees (maximum DBH measured 280.36 cm [measured above the buttress] unlike forests in previous studies where DBH of trees did not exceed 150 cm) (Shaheen et al. 2008, Baishya et al. 2009). Large trees contribute disproportionately to the carbon stock in primary forests (Sist et al. 2014, Hu et al. 2015) and drive variation in aboveground carbon (Slik 2013). As carbon estimates in steep terrain of montane subtropical forests are still underreported (Venter et al. 2017), our results indicate that old‐growth forests with minimal anthropogenic disturbances in this montane region can accumulate substantially high levels of carbon stocks.

### Carbon stock recovery across fallow ages of secondary forest

We found a positive association between fallow period and total carbon stock in regenerating secondary forest, as shown by other studies of recovery in shifting agriculture from tropical forests (Hughes et al. 1999, Read and Lawrence 2003, Pelletier et al. 2012, Chan et al. 2016) and, more generally, by studies of (sub‐) tropical land abandonment (Gilroy et al.2014a, Poorter 2016). Our study also suggests that mature secondary forests reach about one‐half of the levels (56%) of aboveground biomass in old‐growth forest within 30 yr. A similar time frame has been shown in tropical forests of Mexico (Salinas‐Melgoza et al. 2017), Colombia (Gilroy et al. 2014a), and the Brazilian Amazon (D'oliveira et al. 2011).

The exponential increase in total carbon across fallow ages in our study can be influenced by the small‐scale mosaic nature of the shifting cultivation landscape. Close proximity of old‐growth or mature secondary forest to these fallow sites may help animal‐induced seed dispersal (Cole et al. 2010), resulting in increasing rates of forest recovery over time once there has been some regeneration that encourages animals to use the plot. This can create a positive feedback loop with greater recovery leading to increased use by seed dispersing animals that leads to faster recovery. Moreover, regenerating vegetation provides increased protection to the soil from erosion (Tawnenga 1990), which is likely to be particularly important in the study area, which is characterized by steep terrain and high rainfall. Increased protection from erosion is likely to lead to faster recovery, reducing potential for destabilization of young trees.

### Potential of REDD+ in shifting cultivation landscapes

The growing demands for food production with increasing human population have led to either more frequent rotation in existing shifting cultivation systems or expansion of shifting cultivation into old‐growth forest in the tropics (Robichaud et al. 2009). We show that both more frequent cultivation cycles and expansion into old‐growth forest can reduce landscape carbon substantially. These adverse impacts of shifting cultivation make it crucial to implement conservation intervention such as REDD+ for both carbon and biodiversity conservation. Shifting cultivation is likely to have a relatively low opportunity cost of conserving forest under REDD+ as it is a subsistence‐based farming and is mainly practiced in remote regions with limited market access and low crop yields (Borrego and Skutsch 2014). Therefore, REDD+ payments are likely to offset the costs of avoiding deforestation and forest degradation from shifting cultivation at relatively low carbon prices, as found in marginal cattle lands in the Tropical Andes (Gilroy et al. 2014a). This presents an opportunity for REDD+ to provide economically viable financial incentives to effectively manage these landscapes for protecting and enhancing forest carbon stock in shifting cultivation landscapes (Ziegler 2012).

As old‐growth forests are the most important terrestrial carbon sink (Pan 2011) and harbor rich biodiversity (Gibson 2011), including our study area within the Indo‐Malayan global biodiversity hotspot and Eastern‐Himalayan Endemic Bird Area, restricting further expansion of shifting cultivation to such forests would protect significant conservation values. We show that sparing old‐growth forests as protected areas by intensifying cropping in a smaller area (Scenario 3) will be the most optimal strategy under REDD+ for carbon storage. Research from other tropical regions also suggests the importance of sparing old‐growth forest matched within more intensive farming (Gilroy et al. 2014b, Luskin et al. 2017). Given the likely economic viability of REDD+ within shifting cultivation (Mertz 2009), this suggests the potential for substantial biodiversity protection within our biodiverse study region as a free co‐benefit from protecting carbon stocks under REDD+ (Gardner 2012, Gilroy et al. 2014a).

In existing shifting cultivation landscapes without any old‐growth forest, secondary forest creation through regeneration by increasing rotation frequency in a smaller area (Scenario 2) is the next most optimal pathway for REDD+ investment. As regenerating secondary forests store substantial carbon stocks (Bongers et al. 2015) and often harbor rich biodiversity (Gilroy et al. 2014a, Sayer et al. 2017), this could provide co‐benefits for both carbon and biodiversity (Gilroy et al. 2014b, Jantz et al. 2014, Pandey et al. 2014). However, such benefits may change seasonally given that in winter, Himalayan farmland is more diverse than is forest (Elsen et al. 2017). Across the entire cropping area, REDD+ can also provide financial incentives to maintain a relatively longer fallow cycle (baseline scenario with 30‐yr cycle) or transform back from a short to long fallow cycle (5‐ to 10‐, 15‐, or 30‐yr cultivation cycles in Scenario 1) to avoid forest degradation. Such carbon enhancements have shown similar positive outcomes in South‐East Asia, where many countries still prioritize replacing shifting cultivation with alternative land uses (e.g., cash crop plantations) of lower carbon and biodiversity values (Ziegler 2012).

To implement REDD+ within shifting agriculture landscapes in this region, it would be advisable to learn from the Khasi Hill Community REDD+ project (the first REDD+ project in India), which has aimed to reverse deforestation and degradation through forest protection and restoration measures in Meghalaya, northeast India (Sun and Chaturvedi 2016). More generally, India has implemented several policies to reduce deforestation and forest degradation including community forest management, protected area management, and afforestation programs (Murthy et al. 2013), with the Green India Mission focusing on protecting and enhancing both carbon stocks and biodiversity to avert climate change (Ravindranath and Murthy 2010). Learning from the successes and failures of these policies and from established REDD+ readiness activities and protocols (e.g., capacity building and carbon stock assessment) will likely facilitate optimal implementation.

While interpreting the scenario results, it is important to consider two key limitations of this study. First, the scenarios assume that a reduction in cropping area will not reduce crop yield as per hectare yields can be increased by adopting various crop management options. Previous studies from the study region have shown that similar levels of crop yield can be maintained in a smaller area by nutrient supplementation (Tawnenga and Tripathi 1997), optimizing crop choice (Toky and Ramakrishnan 1981), and improved fallow management (Grogan et al. 2012). Second, we did not account for varying opportunity costs of different REDD+ interventions. Although crop yield is assumed to remain constant, other aspects of opportunity costs, such as labor input and timber revenues, may differ depending on whether older fallows or old‐growth forests are spared from shifting cultivation under REDD+ (Scenario 2 or 3 respectively). Similarly, carbon prices may also vary depending on whether existing carbon is saved by avoiding deforestation or degradation (e.g., sparing old‐growth forest in Scenario 3) or enhanced by moving from short to long fallows (e.g., Scenario 1). However, many areas dominated by shifting cultivation are remote for large‐scale timber and crop markets, while prices may vary, it is highly likely that they would remain low compared to the opportunity costs in less remote areas of the tropics (e.g., Borneo [Fisher et al. 2011], Indochina [Warren‐Thomas 2018]). Moreover, protected areas are unlikely to avoid all degradation and deforestation, so protection may reduce carbon loss to a slightly smaller extent than suggested by our models. Any such reductions in carbon savings seem likely to apply similarly to protection of old‐growth and secondary forest. In addition, REDD+ interventions that work effectively with, and are supported by, local communities with an appropriate level of enforcement can be effective in preventing deforestation and degradation (Hayes and Persha 2010, Danielsen 2011).

For successful implementation of REDD+, effective mechanisms to quantify reduction in carbon emission and carbon payments are prerequisites. Moreover, policymakers should also consider information on biodiversity distribution and threats to achieve carbon and biodiversity co‐benefits while prioritizing areas for REDD+ projects (Gardner 2012). It is also important to secure land tenure, reform market policies to create market opportunities for farmers, and to organize training and community activities for active participation of local community in REDD+ (Thrupp et al. 1997).

## Conclusion

Shifting cultivation continues to be widely practiced in many remote montane regions of the (sub‐) tropics, which also harbor much old‐growth forest and biodiversity. The expansion of shifting cultivation into forests and the permanent transition of shifting cultivation into more intensive land‐use systems both drive substantial carbon emissions and biodiversity loss. We suggest explicit pathways for implementing REDD+ to reduce deforestation and forest degradation from shifting cultivation, and successful implementation of these interventions will also likely provide co‐benefits such as biodiversity conservation, provisioning of other ecosystem services, and sustainable rural development (Phelps et al. 2012, Gilroy et al. 2014b, Mukul et al. 2016b). There is thus an urgent need to work with shifting cultivators through capacity building programs to implement these conservation strategies and to enable farmers to meet their production needs in a smaller area of land. Particularly fruitful in generating the income required could be the emerging Bonn Challenge agenda for Forest and Landscape Restoration, and also India's new tax revenue distribution reform (Busch and Mukherjee 2017).

## Data Availability

Data available from Figshare: https://doi.org/10.15131/shef.data.6182147
