# Supplementary material for: Quantifying carbon stocks in shifting cultivation landscapes under divergent management scenarios relevant to REDD+
Source: Ecol Appl. 2018 Jul 25;28(6):1581–93. doi: 10.1002/eap.1764 (PMC6175365; doi:10.1002/eap.1764)
Supplement: Supplementary file 1 [file EAP-28-1581-s001.pdf]

Ecological Applications

Appendix S1- Supplementary information

Quantifying carbon stocks in shifting cultivation landscapes under divergent management scenarios relevant to REDD+

Joli R. Borah, Karl L. Evans, David P. Edwards

*Corresponding author: jborah1@sheffield.ac.uk, +44(0)114 2220123 (Joli R. Borah)

Appendix S1 includes:

Text S1. Changes in carbon stocks 5 years after management changes.

Text S2. Determining the age of secondary forest.

Figure S1. Details of study location and sampling design.

Figure S2. Live and dead carbon accumulation across farmland, secondary forest with age, and old-growth forests.

Figure S3. Difference in landscape level carbon stock under three alternative management regimes of shifting cultivation at the end of 5 years.

Figure S4. Potential pathways for REDD+ investment to protect and enhance forest carbon stocks in a shifting cultivation landscape.

Figure S5. Comparison of total carbon stocks between this study and three previous studies from montane Asia.

Table S1. Study site details and sampling effort.

Table S2. Allometric equations used for biomass estimation of trees, roots and liana sampled.

Table S3. Details of the top three best linear mixed effect models applied to assess change in total, live and dead carbon stock across farmland, secondary and old-growth forest.

Table S4. Details of the top three best linear mixed effect models applied to assess change in total, live and dead carbon stock across fallow ages in secondary forest.

Text S1. Changes in carbon stocks 5 years after management changes

At the end of 5 years, carbon stocks reduced by 18.4%, 30.8% and 71% from the baseline of 2687.9 ± 357.3 Mg/30 ha (mean ± SD) in a 15, 10 and 5-year cycle (Scenario 1.1, 1.2 and 1.3 respectively; Appendix S1: Fig S3) under the business-as-usual scenario of no forest sparing. The second business-as-usual scenario of shifting cultivation expansion (Scenario 4) resulted in 79.6 % loss of the carbon stocks compared to an original old-growth forest landscape (13221.9 ± 1736.2 Mg/30 ha (mean ± SD)).

Landscape carbon increased under REDD+ interventions by secondary forest creation and sparing by 18.3 %, 23% and 26.3% in a 15-year (Scenario 2.1), 10-year (Scenario 2.2) and 5-year cycle (Scenario 2.3) respectively (Appendix S1: Fig. S3). In pioneer shifting cultivation landscapes, only 15.7% carbon stock is lost when intervention is applied by sparing old-growth forest (50%, 66.6% and 83% of the landscape is protected in Scenario 3.1, 3.2 and 3.3 respectively; Appendix S1: Fig. S3). Protecting 50% of old-growth forest whilst the rest of the landscape is managed with a 15-year shifting cultivation cycle (Scenario 3.1; Appendix S1: Fig. S3) reduces carbon loss by 80.3% relative to a landscape managed entirely as shifting cultivation with a 5-year cycle (Scenario 1.3; Appendix S1: Fig. S3).

Text S2. Determining the age of secondary forest

We determined and verified the age of secondary forest regenerating during the fallow period (55 sampling plots) via interviews with members of each village and remote-sensing Landsat images (Landsat 5 TM32, USGS 2017). We conducted semi-structured interviews with famers managing each plot. We interviewed at least five farmers per village (range 5-9) and selected individuals whose age and experience enabled them to provide information on fallow age from first-hand experience. In most villages in this region farmers clear forest patches in groups, so we were able to verify these dates by independently cross-checking with a number of farmers from the same group.

We verified the interview-derived estimates using remote-sensing data to find the year when each sampling plot was last burnt. This is a strong signal of shifting cultivation because fires are always used to clear vegetation, and otherwise are extremely rare in this landscape (personal observation and data from semi-structured interviews). We used Landsat 5 Thematic Mapper (TM) 32-day raw composite images from USGS with band combinations of 7, 4 and 2 – which depicts vegetation as bright green and recently burnt areas as red. These Landsat data were available from 1988 to 2012 and could thus verify precisely the estimates provided by farmers when plots were between three and 28 years old. The difference between interview and Landsat derived estimates ranged from -7 to 9 years (mean absolute difference (± SD) 2.3 ± 2.5 years). Where there were discrepancies in ages between satellite data and interviews, we used the age estimates from satellite data for our analysis. However, for plots cleared after 2012 we used the interview-derived estimates on the assumption that recent memories of clearance dates will be very accurate.

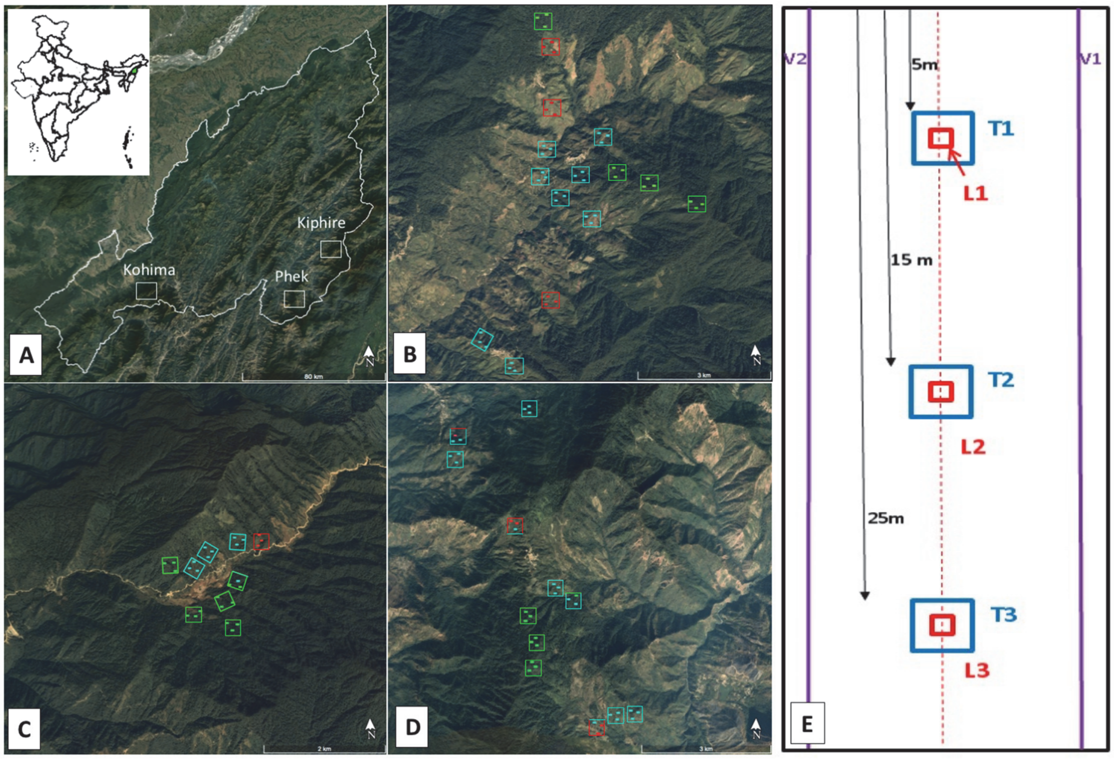

Figure S1. Details of study location and sampling design. The study took place in Nagaland, Northeast India (A), with carbon sampling in a total of 36, 400 m × 400 m squares across three landscapes: Kiphire (B), Kohima (C) and Phek districts (D). Colour of the squares denotes the three habitat types: farmland (red), regenerating secondary forest (blue) and old-growth forest (green). Each sampling square consisted of three 10 m × 30 m plot (E) containing three 2 m^2 sampling sub-plots (T1-3), within each of which there was a 1 m^2 central plot (L1-3), and two 1 m × 30 m sub-plots along the plot margins (V1-2).

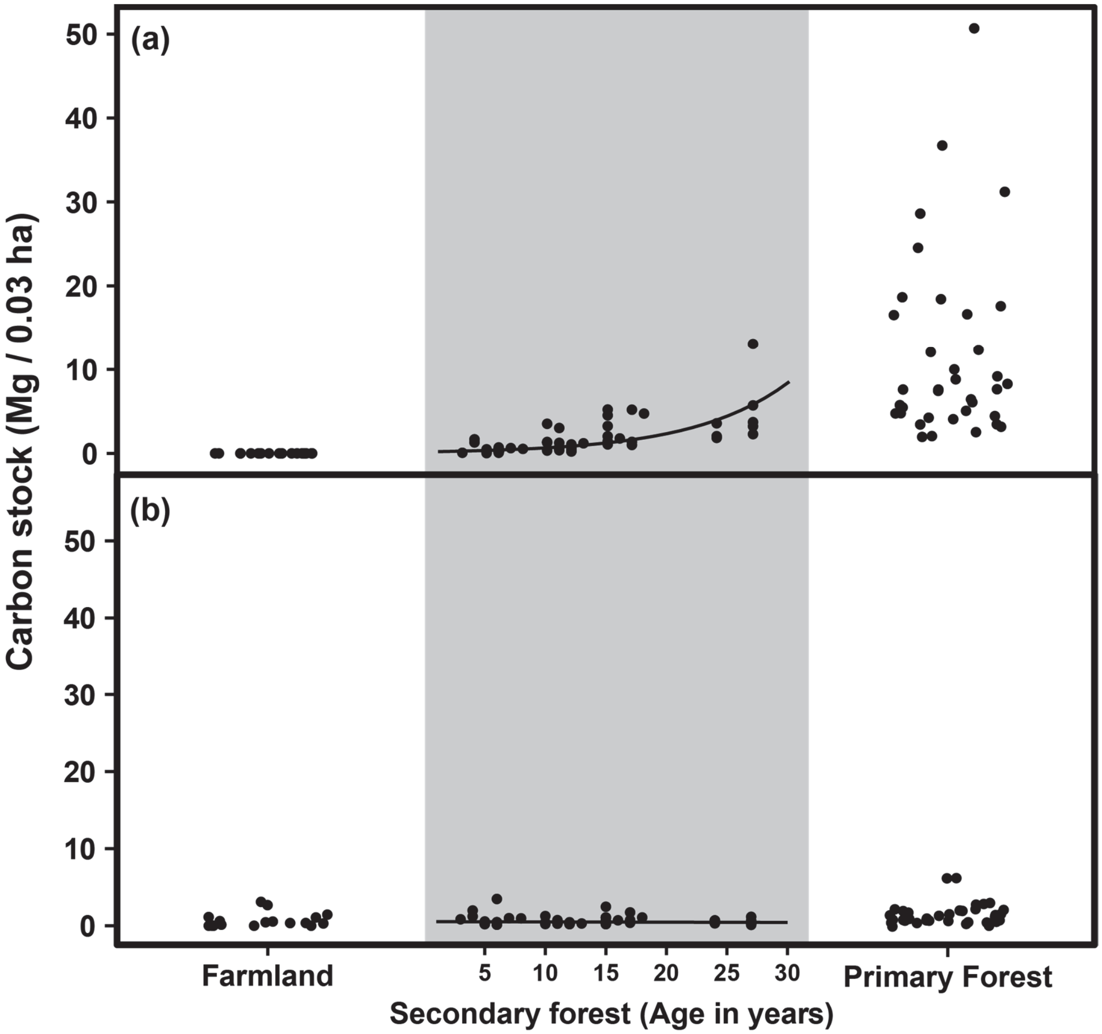

Figure S2. (a) Live and (b) dead carbon accumulation across the three habitats, farmland, secondary forest with age, and old-growth forest plots in Nagaland, Northeast India. Black line in secondary forest (age in years) shows fitted linear mixed effect model.

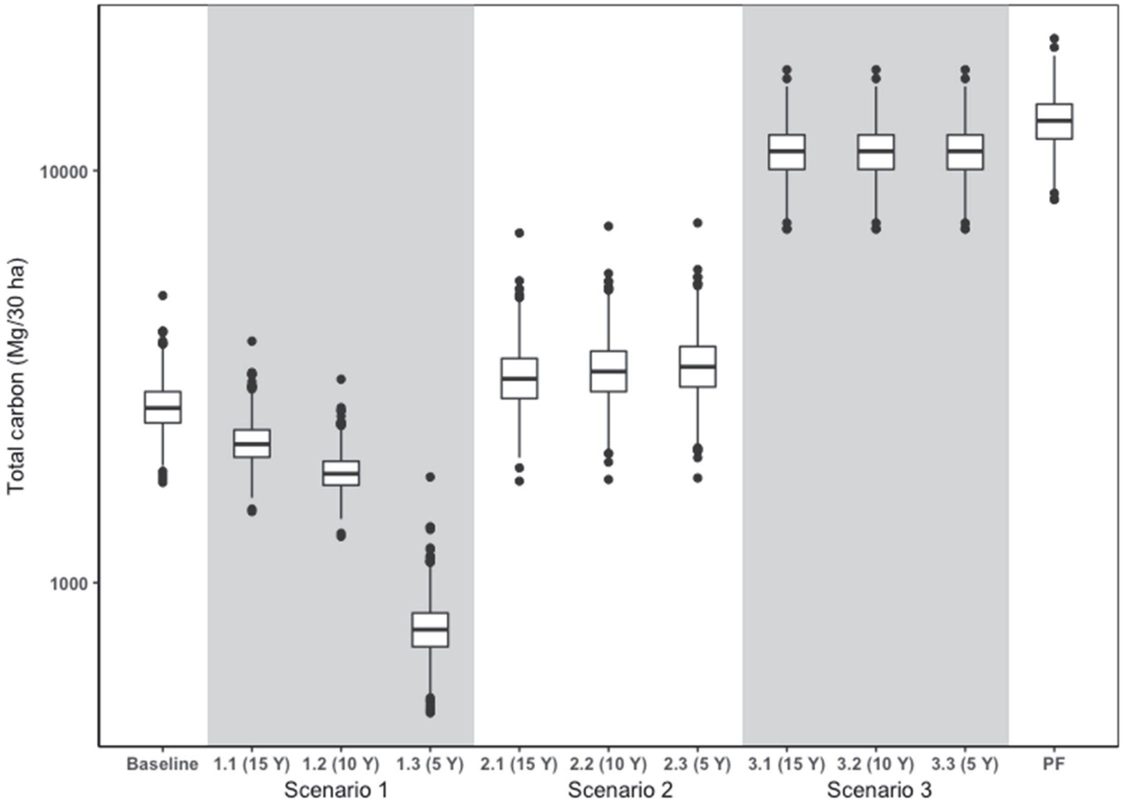

Figure S3. Boxplots showing the difference in landscape level carbon stock under three alternative management regimes of shifting cultivation at the end of 5 years relative to a baseline of 30-year cultivation cycle (Baseline) and old-growth forest landscape (PF) (i)'Business-as-usual' scenario with no forest sparing in Scenario 1 (Scenario 1.1, 15-year cycle; Scenario 1.2, 10-year cycle; Scenario 1.3, 5-year cycle) (ii) REDD+ intervention by secondary forest creation and sparing in Scenario 2 (Scenario 2.1, 15-year cycle; Scenario 2.2,10-year cycle; Scenario 2.3, 5-year cycle) and by restricting shifting cultivation expansion with protection of old-growth forest in Scenario 3 (Scenario 3.1, 15-year cycle; Scenario 3.2,10-year cycle; Scenario 3.3, 5-year cycle).

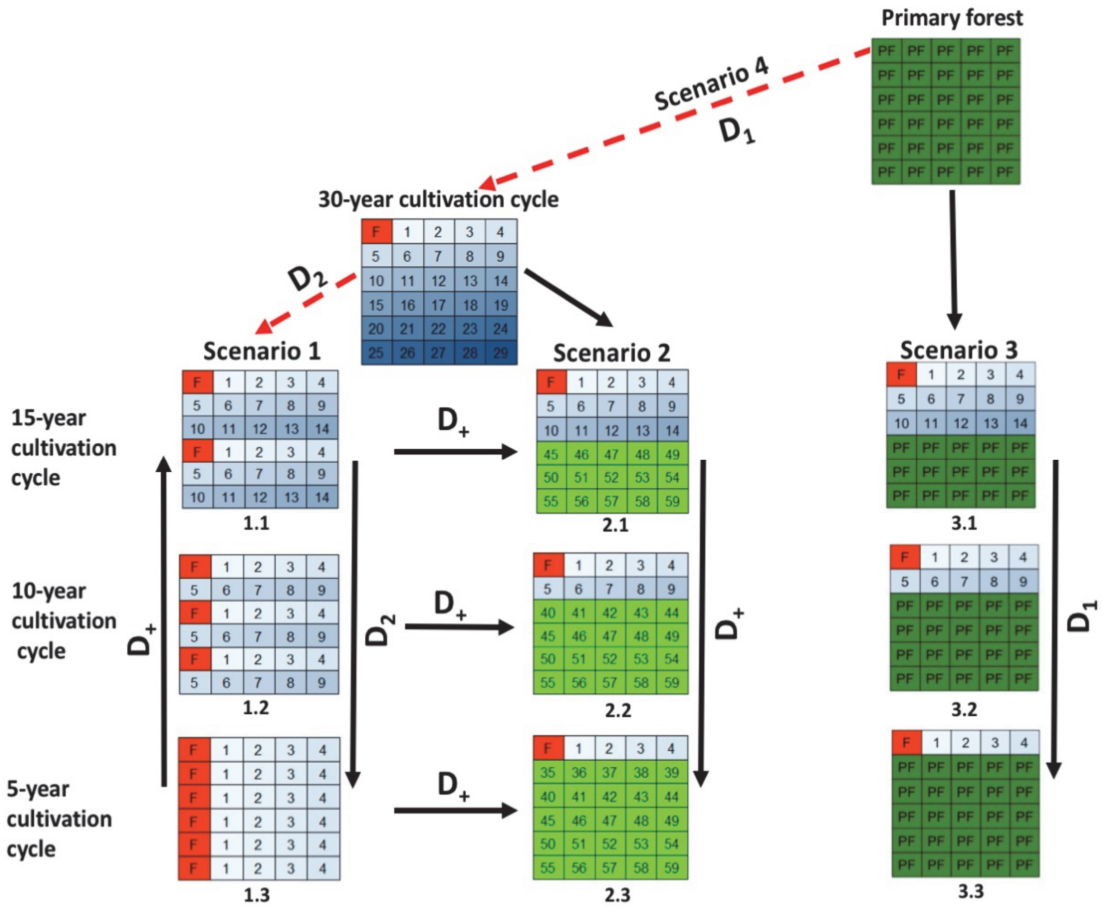

Figure S4. Potential pathways for REDD+ investment to protect and enhance forest carbon stocks in a shifting cultivation landscape under the four sets of management scenarios-avoided deforestation (D_1), avoided forest degradation (D_2) and enhancement of forest carbon (D_+). The scenarios depict no forest sparing (Scenario 1), secondary forest creation and sparing (Scenario 2), new shifting cultivation landscape with sparing old-growth forest (Scenario 3) and shifting cultivation expansion (Scenario 4). Colours indicate habitat types: farmland (F, red), active fallows (1-29 years, different shades of blue), abandoned old fallows (>30 years, light green) and old-growth forests (PF, dark green). Numbers within cells denote the age of the secondary forests.

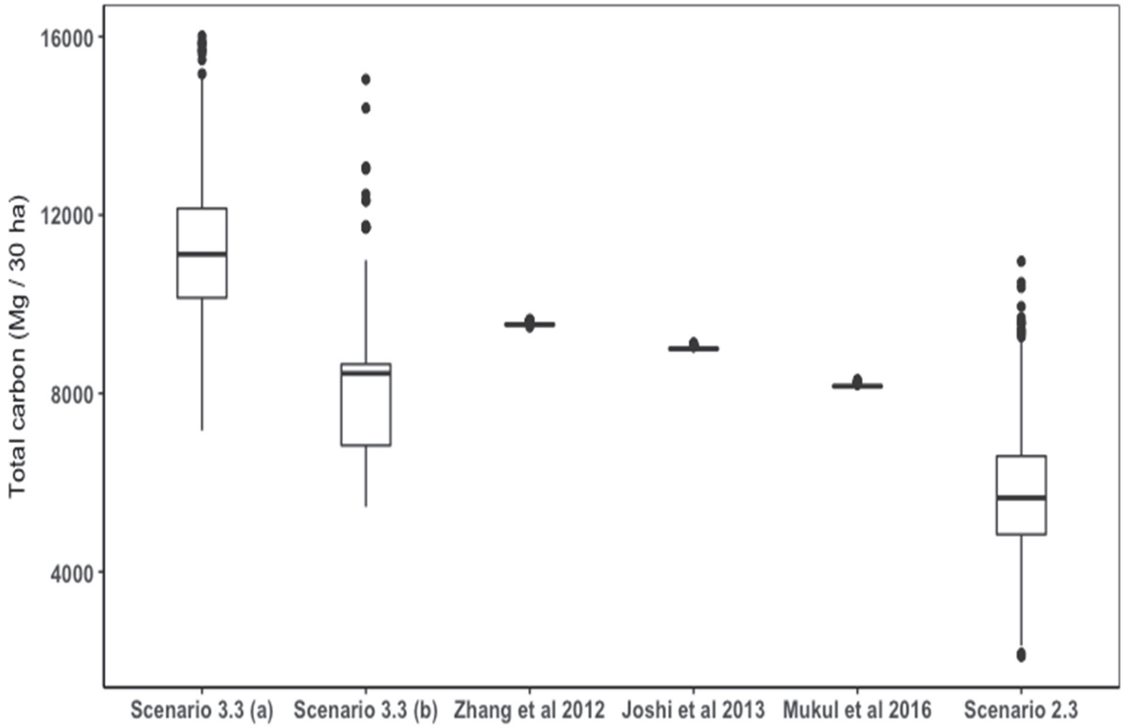

Figure S5. Comparison of total carbon stocks in old-growth forest sparing (Scenario 3.3) and the next best scenario of secondary forest creation and sparing (Scenario 2.3) between this study and three previous studies from montane Asia (Zhang et al. 2013, China; Joshi et al. 2013, India and Mukul et al. 2016, Philippines). Scenario 3.3 (a) and Scenario 3.3 (b) were simulated with mean and median carbon estimates from old-growth forest of this study, respectively.

Table S1. Study site details and sampling effort in Nagaland, Northeast India.

**Landscapes**	**Kiphire**	**Kohima**	**Phek**
Villages	Fakim, Tsundang, Thanamir	Dzuleke	Wazeho, Zhipu, Washelo
Elevation (m)	1722-2652	1716-2001	1487-2309
Location (lat, long)	25°48′18.58″ N 94°58′40.28″ E	25°39′13.76″ N 94°43′31.92″ E	25°36′21.00″ N 94°01′32.43″ E
Sampling points - Farmland	9	3	5
Sampling points - secondary forest	24	10	21
Sampling points - old-growth forest	12	14	10
Total number of sampling points	45	27	36

Table S2. Allometric equations used for biomass estimation of trees, roots and liana sampled in Nagaland, Northeast India; where, AGB=Above ground biomass for individual tree, DBH=diameter at breast height, ρ=wood specific gravity, E= (0.178*TS-0.938* CWD-6.61*PS)*10^{-3}, E-measure of environmental stress, TS- temperature seasonality, CWD-climatic water deficit, PS-precipitation seasonality, diameter at 30 cm=1.235 DBH+0.002 $(DBH)^2$ and BA-Basal area

Component	Source	Region	Equation	Sample size
Trees, old-growth forest	Dung et al.2012	Vietnam	AGB = 0.3429 $(DBH)^{2.3028}$ $(\rho)^{1.2901}$	201
	Chave et al.2014	Pantropical	AGB= exp [-1.803-0.976 E + 0.976 (log ρ) + 2.673 (log DBH) -0.0299$(\log DBH)^2$]	
Trees, Secondary forest	Ketterings et al.2001	Indonesia	AGB= exp [-2.207 + 2.62 (ln DBH) + (ln ρ)]	29
	Van Breugel et al.2011	Central America	AGB = exp [-1.130+ 2.267 (ln DBH) + 1.186 (ln ρ)]	244
	Chave et al.2014	Pantropical	AGB=exp [-1.803-0.976 E+0.976 (ln ρ) + 2.673 (ln DBH) -0.0299 $(\ln DBH)^2$]	4004
Liana	Putz 1983	Amazon	AGB = exp [0.12+0.91 log (BA)]	17
	Gehring et al.2005	Amazon	AGB = exp [-7.114+2.276 ln (diameter at 30 cm)]	561
	Schnitzer et al.2006	S. America	AGB=exp [-1.484+2.657 (ln DBH)]	424
	Sierra et al.2007	Colombia	AGB= exp [0.028+1.841 (ln DBH)]	33
	Addo-Fordjour and Rahmad 2013	Malaysia	AGB=0.262+1.934 (DBH)	60

Table S3. Details of the top three best models based on AICc values obtained from linear mixed effect model applied to assess change in total, live and dead carbon stock across the three habitats, i.e. farmland, secondary and old-growth forest

Model	Habitat	Elevation	Habitat * Elevation	df	Log Likelihood	AICc	delta	weight	Marginal R2	Conditional R2
Lmer (log10(Total carbon) ~ Habitat + (1| Landscape/Square)	+	NA	NA	6	-54.74	122.3	0.00	0.59	0.57	0.76
Lmer (log10(Total carbon) ~ Habitat + Elevation + (1|Landscape/Square)	+	0.09	NA	7	-54.24	123.6	1.28	0.31	0.57	0.76
Lmer (log10(Total carbon) ~ Habitat + Elevation + (1|Landscape/Square) + Age*Elevation	+	0.19	+	9	-53.09	126.0	3.72	0.09	0.59	0.75
Lmer (log10(Live carbon) ~ Habitat + Elevation+ Habitat* Elevation+ (1| Landscape/Square)	+	0.07	+	9	-49.57	119	0.00	0.49	0.81	0.85
Lmer (log10(Live carbon) ~ Habitat + (1| Landscape/Square)	+	NA	NA	6	-53.39	119.6	0.65	0.36	0.79	0.85
Lmer (log10(Live carbon) ~ Habitat + Elevation + (1| Landscape/Square)	+	0.04	NA	7	-53.18	121.5	2.50	0.14	0.79	0.85
Lmer (log10(Dead carbon) ~ Habitat + (1| Landscape/Square)	+	NA	NA	6	-46.01	104.9	0.00	0.72	0.19	0.65
Lmer (log10(Dead carbon) ~ Habitat + Elevation+ (1| Landscape/Square)	+	0.001	NA	7	-46.01	107.2	2.29	0.23	0.19	0.65
Lmer (log10(Dead carbon) ~ Habitat +Elevation+ Habitat * Elevation+ (1| Landscape/Square)	+	-0.21	+	9	-45.67	111.2	6.32	0.03	0.20	0.67

Table S4. Details of the top three best models based on delta AIC values obtained from linear mixed effect model applied to assess change in total, live and dead carbon stock fallow ages in secondary forest.

Model	Age	Elevation	Age*Elevation	df	Log Likelihood	AICc	delta	weight	Marginal R2	Conditional R2
Lmer (log10 (Total carbon) ~ Age + (1|Landscape/Square)	0.589	NA	NA	5	- 6.948	25.1	0.00	0.728	0.374	0.640
Lmer (log10 (Total carbon) ~ Age + Elevation + (1|Landscape/Square)	0.588	-0.019	NA	6	-6.936	27.6	2.50	0.208	0.378	0.638
Lmer (log10 (Total carbon) ~ Age + Elevation + (1|Landscape/Square) + Age*Elevation	0.636	-0.122	0.135	7	-6.813	30.0	4.89	0.063	0.379	0.634
Lmer (log10 (Live carbon) ~ Age + (1| Landscape/Square)	0.71	NA	NA	5	-21.79	54.8	0.00	0.629	0.51	0.65
Lmer (log10 (Live carbon) ~ Age+ Elevation + (1| Landscape/Square)	0.69	-0.15	NA	6	21.33	56.4	1.62	0.280	0.53	0.65
Lmer (log10 (Live carbon) ~ Age + Elevation + Age* Elevation+ (1| Landscape/Square)	0.75	-0.25	0.17	7	-21.14	58.7	3.86	0.091	0.54	0.65
Lmer (log10 (Dead carbon) ~ (1| Landscape/Square)	NA	NA	NA	4	-17.29	43.4	0.00	0.56	0.00	0.49
Lmer (log10 (Dead carbon) ~ Age + (1| Landscape/Square)	-0.06	NA	NA	5	17.17	45.6	2.17	0.19	0.01	0.51
Lmer (log10 (Dead carbon) ~ Elevation + (1| Landscape/Square)	NA	0.06	NA	5	-17.24	45.7	2.31	0.18	0.00	0.49

LITERATURE CITED

Addo-Fordjour, P., and Z. B. Rahmad. 2013. Development of allometric equations for estimating above-ground liana biomass in tropical primary and secondary forests, Malaysia. *International Journal of Ecology* Doi:10.1155/2013/658140

Chave, J., M. Réjou-Méchain, A. Búrquez, E. Chidumayo, M. S. Colgan, W. B. C. Delitti, A. Duque, T. Eid, P. M. Fearnside, R. C. Goodman, M. Henry, A. Martínez-Yrízar, W. a. Mugasha, H. C. Muller-Landau, M. Mencuccini, B. W. Nelson, A. Ngomanda, E. M. Nogueira, E. Ortiz-Malavassi, R. Pélissier, P. Ploton, C. M. Ryan, J. G. Saldarriaga, and G. Vieilledent. 2014. Improved allometric models to estimate the aboveground biomass of tropical trees. *Global Change Biology* **20** :3177–3190.

Dung, N. T., P. M. Toai, V. T. Hung, L. T. Anh, and P. V. Khoa. 2012. Tree allometric equations in evergreen broadleaf and bamboo forests in the North Central coastal region, Viet Nam. In: Inoguchi, A., Sola, G., Henry, M., Birigazzi, L. (Eds.), Tree Allometric Equation Development for Estimation of Forest Above-Ground Biomass in Viet Nam. Hanoi, Viet Nam.

Gehring, C., M. Denich, and P. L. G. Vlek. 2005. Resilience of secondary forest regrowth after slash-and-burn agriculture in central Amazonia. *Journal of Tropical Ecology* **21**:519–527.

Joshi, N. R., A. Tewari, and D. B. Chand. 2013. Impact of Forest fire and aspect on phytosociology, tree biomass and carbon stock in Oak and Pine mixed Forests of Kumaun central Himalaya, India. *Researcher* **5**:1-8.

Ketterings, Q. M., R. Coe, M. Van Noordwijk, Y. Ambagau', and C. a. Palm. 2001. Reducing uncertainty in the use of allometric biomass equations for predicting above-ground tree biomass in mixed secondary forests. *Forest Ecology and Management* **146**:199–209.

Mukul, S. A., J. Herbohn, and J. Firn. 2016. Tropical secondary forests regenerating after shifting cultivation in the Philippines uplands are important carbon sinks. *Scientific Reports* **6**:1–12.

Putz, F. E. . 1983. Liana Biomass and Leaf Area of a " Tierra Firme " Forest in the Rio Negro Basin , Venezuela. *Biotropica* **15**:185–189.

Schnitzer, S. A., S. J. DeWalt, and J. Chave. 2006. Censusing and measuring lianas: A quantitative comparison of the common methods. *Biotropica* **38**:581–591.

Sierra, C. A., J. I. del Valle, S. A. Orrego, F. H. Moreno, M. E. Harmon, M. Zapata, G. J. Colorado, M. A. Herrera, W. Lara, D. E. Restrepo, L. M. Berrouet, L. M. Loaiza, and J. F. Benjumea. 2007. Total carbon stocks in a tropical forest landscape of the Porce region, Colombia. *Forest Ecology and Management* **243**:299–309.

van Breugel, M., J. Ransijn, D. Craven, F. Bongers, and J. S. Hall. 2011. Estimating carbon stock in secondary forests: Decisions and uncertainties associated with allometric biomass models. *Forest Ecology and Management* **262**:1648–1657.

Zhang, Y., F. Gu, S. Liu, Y. Liu, and C. Li. 2013. Variations of carbon stock with forest types in subalpine region of southwestern China. *Forest Ecology and Management* **300**:88-95.
